# Next Generation Molecular Diagnosis of Hereditary Spastic Paraplegias: An Italian Cross-Sectional Study

**DOI:** 10.3389/fneur.2018.00981

**Published:** 2018-12-04

**Authors:** Angelica D'Amore, Alessandra Tessa, Carlo Casali, Maria Teresa Dotti, Alessandro Filla, Gabriella Silvestri, Antonella Antenora, Guja Astrea, Melissa Barghigiani, Roberta Battini, Carla Battisti, Irene Bruno, Cristina Cereda, Clemente Dato, Giuseppe Di Iorio, Vincenzo Donadio, Monica Felicori, Nicola Fini, Chiara Fiorillo, Salvatore Gallone, Federica Gemignani, Gian Luigi Gigli, Claudio Graziano, Renzo Guerrini, Fiorella Gurrieri, Ariana Kariminejad, Maria Lieto, Charles Marques LourenḈo, Alessandro Malandrini, Paola Mandich, Christian Marcotulli, Francesco Mari, Luca Massacesi, Maria A. B. Melone, Andrea Mignarri, Roberta Milone, Olimpia Musumeci, Elena Pegoraro, Alessia Perna, Antonio Petrucci, Antonella Pini, Francesca Pochiero, Maria Roser Pons, Ivana Ricca, Salvatore Rossi, Marco Seri, Franco Stanzial, Francesca Tinelli, Antonio Toscano, Mariarosaria Valente, Antonio Federico, Anna Rubegni, Filippo Maria Santorelli

**Affiliations:** ^1^Molecular Medicine, Pisa, Italy; ^2^Department of Biology, University of Pisa, Pisa, Italy; ^3^Department of Medical and Surgical Sciences and Biotechnologies, University of Rome Sapienza, Latina, Italy; ^4^Department of Medicine, Surgery and Neurosciences, Medical School, University of Siena, Siena, Italy; ^5^Department of Neurosciences, Reproductive and Odontostomatologic Sciences, Federico II University, Naples, Italy; ^6^IRCCS Fondazione Policlinico Universitario A. Gemelli, Rome, Italy; ^7^Institute of Neurology, Catholic University of Sacred Heart, Rome, Italy; ^8^Department of Pediatrics, Institute for Maternal and Child Health-IRCCS Burlo Garofolo, Trieste, Italy; ^9^Genomic and Post-Genomic Center, IRCCS Mondino Foundation, Pavia, Italy; ^10^Second Division of Neurology, Department of Medical, Surgical, Neurological, Metabolic and Aging Sciences, University of Luigi Vanvitelli, Naples, Italy; ^11^IRCCS Istituto delle Scienze Neurologiche di Bologna-UOC Clinica Neurologica, Bologna, Italy; ^12^Istituto delle Scienze Neurologiche di Bologna-UOC Neuropsichiatria Infantile, Bologna, Italy; ^13^Department of Neurosciences, Sant'Agostino-Estense Hospital, Azienda Ospedaliero Universitaria di Modena, Modena, Italy; ^14^Pediatric Neurology and Neuromuscular Disorders, University of Genoa and Istituto Giannina Gaslini, Genova, Italy; ^15^Neurology I, Department of Neuroscience and Mental Health, AOU Città della Salute e della Scienza, Turin, Italy; ^16^Neurology Clinic, Azienda Ospedaliero Universitaria Santa Maria della Misericordia, Udine, Italy; ^17^Medical Genetics Unit, Sant'Orsola-Malpighi University Hospital, Department of Medical and Surgical Sciences, University of Bologna, Bologna, Italy; ^18^Pediatric Neurology Unit, Children's Hospital A. Meyer, University of Firenze, Florence, Italy; ^19^Institute of Genomic Medicine, Catholic University of the Sacred Heart, Rome, Italy; ^20^Clinical Genetics, Kariminejad-Najmabadi Pathology & Genetics Research Center, Tehran, Iran; ^21^Neurogenetics Division, Clinics Hospital of Ribeirão Preto, University of São Paulo, São Paulo, Brazil; ^22^Department of Neurosciences, Rehabilitation, Ophthalmology, Genetics, Maternal and Child Health, Section of Medical Genetics, University of Genoa, Genoa, Italy; ^23^Medical Genetics Unit, Department of Diagnosis, Pathology and Treatments of High Technological Complexity, IRCCS Ospedale Policlinico San Martino, Genoa, Italy; ^24^Department of Neurosciences Drugs and Child Health, University of Florence, Florence, Italy; ^25^Child Neuropsychiatry, ULSS 7 Pedemontana, Vicenza, Italy; ^26^Department of Clinical and Experimental Medicine, University of Messina, Messina, Italy; ^27^Department of Neurosciences, University of Padua, Padua, Italy; ^28^Neurology Department, San Camillo Hospital, Rome, Italy; ^29^Metabolic and Muscular Unit, Neuroscience Department, Meyer Children's Hospital, Florence, Italy; ^30^First Department of Pediatrics, Aghia Sophia Children's Hospital, University of Athens, Athens, Greece; ^31^Clinical Genetics Service and South Tyrol Coordination Center for Rare Diseases, Department of Pediatrics, Regional Hospital of Bolzano, Bolzano, Italy

**Keywords:** hereditary spastic paraplegia, next generation sequencing, neurogenetics, diagnostic yield, variants of unknown significance

## Abstract

Hereditary spastic paraplegia (HSP) refers to a group of genetically heterogeneous neurodegenerative motor neuron disorders characterized by progressive age-dependent loss of corticospinal motor tract function, lower limb spasticity, and weakness. Recent clinical use of next generation sequencing (NGS) methodologies suggests that they facilitate the diagnostic approach to HSP, but the power of NGS as a first-tier diagnostic procedure is unclear. The larger-than-expected genetic heterogeneity—there are over 80 potential disease-associated genes—and frequent overlap with other clinical conditions affecting the motor system make a molecular diagnosis in HSP cumbersome and time consuming. In a single-center, cross-sectional study, spanning 4 years, 239 subjects with a clinical diagnosis of HSP underwent molecular screening of a large set of genes, using two different customized NGS panels. The latest version of our targeted sequencing panel (*SpastiSure3.0*) comprises 118 genes known to be associated with HSP. Using an in-house validated bioinformatics pipeline and several *in silico* tools to predict mutation pathogenicity, we obtained a positive diagnostic yield of 29% (70/239), whereas variants of unknown significance (VUS) were found in 86 patients (36%), and 83 cases remained unsolved. This study is among the largest screenings of consecutive HSP index cases enrolled in real-life clinical-diagnostic settings. Its results corroborate NGS as a modern, first-step procedure for molecular diagnosis of HSP. It also disclosed a significant number of new mutations in ultra-rare genes, expanding the clinical spectrum, and genetic landscape of HSP, at least in Italy.

## Introduction

Hereditary spastic paraplegia (HSP) is a term used to refer to a group of rare (about 1.8 individuals per 100,000) (1) genetically heterogeneous neurodegenerative motor neuron disorders characterized by progressive age-dependent loss of corticospinal motor tract function, leading to lower limb spasticity, urinary bladder dysfunction, and weakness. Next generation sequencing (NGS) methods have recently emerged as the best approach for the genetic study of HSP (2), having allowed, over the past 4 years, the identification of more than 10 novel causative *SPastic Gait* (*SPG*) genes. To date, 85 different spastic gait disease loci, and 79 known causative *SPG* genes, have been identified (3, 4).

Although it is relatively easy, in appropriate clinical practice settings, to reach probable or possible clinical diagnoses of HSP, the high levels of genetic heterogeneity and different patterns of inheritance associated with this group of disorders make molecular diagnosis challenging. NGS, with its innovative technology, is a rapid, high-throughput and cost-effective approach for identifying the genetic background of Mendelian disorders. Target resequencing multigene panels (TRPs) represents the most cost-effective approach involving analysis of the coding exons of a restricted number of genes. Additional high-throughput NGS methods are whole exome sequencing (WES), covering the full set of DNA sequences encoding and whole genome sequencing (WGS) representing the most expensive, all-inclusive technique (5). However, the true informativeness and diagnostic power of these techniques in clinical settings is often limited due to difficulties in processing the considerable amount of information generated through deep sequencing, as well as imperfect genotype-phenotype correlations.

To ascertain the effective diagnostic power of NGS in a real-life neurogenetic setting, we carried out a cross-sectional study adopting a targeted resequencing gene panel (TRP) method already validated in research studies on HSP (6). Although sequencing a whole-exome (or the full genome) provides unparalleled genetic information, we reasoned that panel-based sequencing offers some advantages owing to cost savings and the ease and speed of data interpretation allowing more rapid translation at bedside. The study involved 239 consecutive patients presenting clinical signs of HSP, recruited over the past 4 years (September 2014–August 2018) in tertiary neurological or neuropediatric centers in Italy; blood samples were analyzed at a single center. In reporting the results of this study, we describe different rates of molecular diagnosis in HSP, and discuss the advantages and disadvantages of our strategy as a first- or second-tier approach in the diagnostic workflow of motor neuron disorders, illustrating some unexpected results as well as major limitations.

## Materials and Methods

### Patients and Study Design

With the help of the Italian Spastic Paraplegias and Ataxias Network (ITASPAX), we consecutively collected DNA samples from patients with a clinical diagnosis of spastic paraplegia. Each index case underwent a detailed neurological examination, carried out by a neurologist or neuropediatric specialist at the clinical center to which the patient in question had been referred. This examination included application of the Spastic Paraplegia Rating Scale (SPRS) (7). Phenotypes were classified as pure HSP or complicated HSP according to the Harding criteria (8). Genes universally recognized to be responsible for HSP (Table S1) were investigated using a customized NGS TRP strategy described elsewhere (6). The recruitment of patients (*n* = 239) and collection of blood samples were performed between September 2014 and August 2018. Whenever possible, major clinical and demographic characteristics, presumed age at disease onset, disease duration and disease severity (SPRS) scores were recorded using a common clinical investigation proforma (case report form, CRF); these data were subsequently used for correlations with genes/variants identified in the study. For cross-sectional analyses of disease severity, the first documented SPRS score in each proband was selected, whereas all available SPRS scores were included for longitudinal analyses.

### Standard Protocol Approvals, Registrations, and Consent

All the participants, including relatives involved in segregation studies, provided written informed consent in accordance both with Italian National Health System guidelines and with the Declaration of Helsinki. This consent was collected by the various clinical centers belonging to the ITASPAX network. The study was approved by the Tuscany Regional Pediatric Ethics Committee and also by the Ethics in Research Committee of IRCCS Fondazione Stella Maris (Pisa, Italy). Storage/handling of genetic and personal data complied with Italian National Health Institute (ISS) regulations on ethical and biomedical research and with relevant current legislation.

### Targeted Sequencing Workflow and Sequencing Analysis

Genomic DNA was extracted from peripheral venous blood using the MagPurix Blood DNA Extraction Kit 200 designed for the MagPurix DNA Extractor (Zinexts, Taiwan).

**Figure 1:**
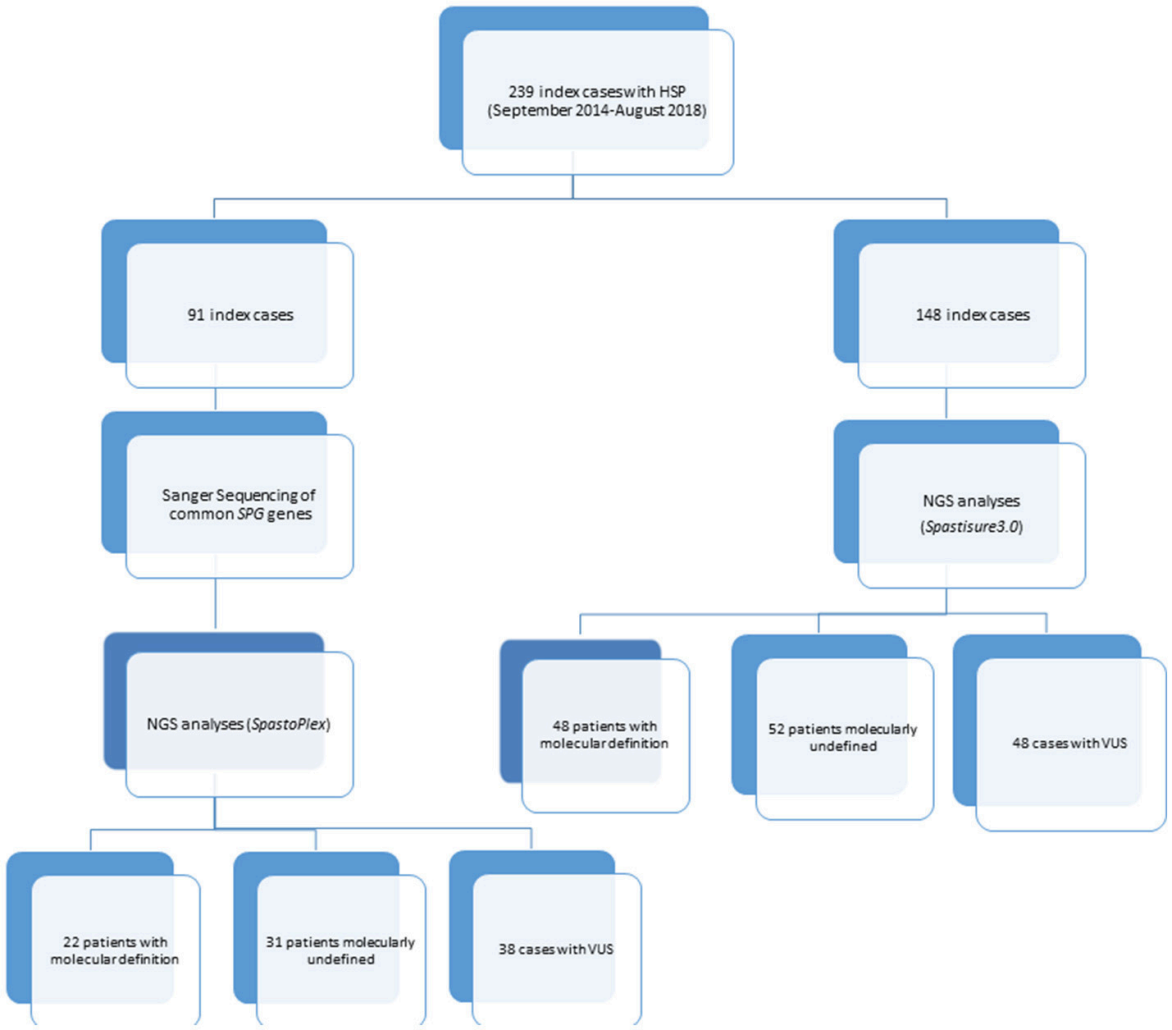
Flowchart showing design study.

A library probe was then prepared according to the manufacturer's instructions (Agilent Technologies, Santa Clara, CA, United States), and the 2200 TapeStation Assay kit (Agilent Technologies) was used to validate and quantify this library preparation. Following the manufacturer's recommendations, two different customized TRPs were designed over the course of this study. One of them, termed *Spastoplex*, contained 72 genes and was designed using Haloplex technology (Agilent Technologies) for the Illumina Sequencing system (Illumina Inc., San Diego, CA); it was employed as a second-/third-tier test in 91 index cases in whom absence of punctate mutations and gene deletions/duplications in *SPAST*/SPG4, *ATL1*/SPG3A, *REEP1*/SPG31, *SPG7, CYP7B1*/SPG5, and *KIAA1840*/SPG11 had already been documented (even by other laboratories) (9, 10). The other TRP (*SpastiSure3.0*) was designed using SureSelect QXT technology (Agilent Technologies) (11), and contained all 118 genes thus far associated with HSP (latest update 02/18); it was used as first-tier diagnostic approach in the other 148 consecutive index cases (see Figure 1). Both TRPs had a mean coverage of 99.66%, ensuring an at least 100X read depth of the targeted regions.

Sequencing was performed on a dedicated platform where 24 samples were run in a single lane of a NextSeq500 system (Illumina), generating 150-bp paired end reads to obtain sequenced clusters passing filter >90% and an average coverage of at least 250X.

### Variant and Bioinformatics Analysis

Raw data (i.e., the generated *fastq* files in each data run) were analyzed using SureCall3.0 software (Agilent) and converted into *vcf* format. Annotation of variants was performed using the open-source wANNOVAR software (wannovar.wglab.org/) and the Ingenuity Variant Analysis web-based application (Qiagen, Hilden, Germany) using a standard alignment and calling protocol [see 6]. In view of the high number of alterations identified in each patient, bioinformatic filters were applied to prioritize type of mutation (missense, in/del, stop gain, or stop loss), frequency in public and in in-house polymorphic databases, and annotation as disease-associated variants according to a pipeline already validated in a research setting (6). Specifically, variants were filtered in such a way as to exclude low affinity ones (coverage ≥30X) and those located in deep intronic or untranslated regions. Synonymous variants were filtered out unless they predicted alterations in exonic splice enhancers. All variants were also filtered to retain only those with minor allele frequency (MAF ≤1% for autosomal recessive and ≤ 0.2% for autosomal dominant transmission) in the 1000G database (http://www.1000genomes.org/, last accessed 08/2018), the dbSNP database version 146 (http://www.ncbi.nlm.nih.gov/SNP/, accessed 08/ 2018), the ESP6500SI-V2 database (evs.gs.washington.edu/, 12/2017), and the ExAC 3.0 browser dataset (exac.broadinstitute.org, 08/2018), and also for number of homozygotes as listed in gnomAD (gnomad.broadinstitute.org/, 08/2018), in order to retain those with 0 or 1 homozygotes, or for which this data is not reported. The sole exception to this latter criterion was the c.1529C>T, p.Ala510Val variant in *SPG7*, which is reported to be pathogenic despite showing a relatively high number of homozygotes (*n* = 2, allele frequency 0.002) in the European population (see gnomad.broadinstitute.org/variant/16-89613145-C-T).

Variants were classified according to the published guidelines of the American College of Medical Genetics and Genomics (12) and pathogenicity was examined *in silico* using at least the following seven prediction tools: Polyphen2 (genetics.bwh.harvard.edu/pph2), SIFT (sift.jcvi.org/), UMD-Predictor (umd-predictor.eu ), VEST missense score (https://www.cravat.us), and GERP RS score (https://varsome.com/search-results/GERP), which were used to evaluate missense variants, and Human Splicing Finder v3.1 (www.umd.be/HSF3) and BDGP: Splice Site Prediction by Neural Network (www.fruitfly.org/seq_tools/splice.html), which were used to investigate those variants that could affect splicing. We retained missense variants that were predicted to be damaging by at least 4 of 5 tools, providing they respected our filters. Presence of the specific variant in the Human Genome Mutation Database (HGMD, www.hgmd.cf.ac.uk/) was not considered a mandatory criterion for attributing pathogenic significance.

### String Network

In each patient, variants of unknown significance (VUS) associated with clear pathogenic changes were further investigated using STRING (https://string-db.org) in order to highlight possible functional protein-protein interactions.

### Confirmation of Variants

Putatively deleterious variants were validated by PCR-based standard capillary Sanger sequencing, both in probands and in relatives available for segregation studies, also to determine whether the mutations were inherited or occurred *de novo*. Segregation analyses contributed to the definition of pathogenic variants. Indeed, whenever more than one change had been prioritized in the index case, study of affected (*n* = 113 individuals) and unaffected relatives (*n* = 124) helped us to determine which variants were more likely to be disease causative.

### MLPA Testing

Multiplex ligation-dependent probe amplification (MLPA) was always performed to detect gene deletions/duplications in *SPAST*/SPG4 (13, 14), and in cases that also presented a single mutation in a relatively frequent recessive gene (*SPG7, SACS* or *KIAA1840*/SPG11), in order to detect possible second mutations. We used the commercially available Salsa Kits P165-C2 (for *SPAST*/SPG4 and *ATL1*/SPG3A), P213-B2 (for *REEP1*/SPG31 and *SPG7*), P306-B1 (for *KIAA1840*/SPG11), and P441-A1 (for *SACS*), according to the manufacturer's instructions (MRC-Holland, Amsterdam). MLPA results were analyzed using Coffalyser v.140721.1958 software (MRC-Holland, Amsterdam).

## Results

### Clinical Findings

Over a 4-year period, we collected 476 samples: 239 from index patients (median age at latest examination 35 years, range 1–82 years), and 237 from patients' affected or unaffected relatives. The average age at disease onset in the entire group of investigated patients was 18 years ± 18.7 (SD), and the vast majority of the cases were Italians with Italian parents. Around half of the cases were men (131/239, 55%) and 72% were adults (>16 years).

Predictably pathogenic mutations were identified in 70 index patients (34 men and 36 women), whose clinical information is summarized in Table 1. Of these patients, assigned a positive molecular diagnosis, 23% showed a dominant pattern of inheritance and 27% clear recessive inheritance; the largest proportion (44%) comprised apparently sporadic patients, while X-linked forms accounted for the smallest proportion (6%, 4 patients). Complex forms were more frequent than pure HSP (65 vs. 35%) and they were more common among sporadic or autosomal recessive (AR) HSP cases, as also reported by others (3).

**Table 1:** Clinical features in patients with molecular diagnostic confirmation.

M/F	34/36
Familial/sporadic	39/31
Dominant/recessive/X-linked	16/19/4
Age at onset, y, mean ± SD (*n*)	17.5 ± 18.7 (56)
Duration, mean ± SD (*n*)	20.3 ± 1.2 (59)
Disability, mean ± SD (*n*)	2.4 ± 1.6 (63)
SPRS, average score (*n*)	18.5 (48)
**DISABILITY**	
Stick use	62.9%
Wheelchair use	28.5%
**UPPER LIMB**	
Hypertonia	25.5%
Hyperreflexia	24.5%
**LOWER LIMB**	
Abnormal vibration sense	68.2%
Amyotrophy	9.5%
*Pes cavus*	26.9%
Bilateral clonus	71.4%
Urinary dysfunction	61.7%
**ABNORMAL MEP**	
Lower limbs	73.2%
Upper limbs	55.5%
**BRAIN IMAGING FEATURES**	
MRI abnormalities	68%
Cerebellar atrophy	22%
Hyperintense WM	12%
Thin corpus callosum	9%

*SPRS, Spastic Paraplegia Rating Scale; WM, white matter*.

The patients with a conclusive diagnosis had an average age at disease onset of 17.5 years ± 18.7 (SD) years with a disease duration of 20.3 (1.2) years at first examination. The time to diagnosis was calculated as the months between the appearance of the first medical sign requiring neurological evaluation and molecular diagnostic confirmation. This time could be ascertained with certainty in only 28 patients, all of whom underwent *SpastiSure3.0* analysis, and it was, on average, 25.4 months (range: 5.2–37.8).

The clinical presentation of HSP was found to be heterogeneous. Whilst lower limb spasticity was detected in almost all the patients with a clear molecular diagnosis of HSP, brisk reflexes were found in 75%, and bilateral clonus in about 70%. Sensory and cerebellar ataxias were rarely observed (33%), whereas deep sensation was impaired in 68%. Neurophysiological investigations were significantly impaired in 75% of the cases and urinary sphincter disturbances were recorded in about 62%. Abnormal brain MRI findings were seen in 49% of the 70 molecularly defined cases, mostly consisting of cerebellar atrophy or thinning of the corpus callosum, or both. Spine MRI abnormalities were documented in 15% of the index cases. Behavioral abnormalities, including panic disorder, major depression, substance abuse and attention deficit hyperactivity disorder, were observed in five cases. Other co-morbidities included cluster headache (*n* = 2), optic atrophy (*n* = 1), lower motor neuron disease (*n* = 3), early ovarian failure (*n* = 3), myoclonic seizures (*n* = 2), and grand mal seizures (*n* = 1). Disease severity was ascertained on the basis of disability scores (where 1 = normal, 2 = walks but cannot run, 3 = walks with aids, 4 = wheelchair bound), and corresponded to an average score of 2.6, with less than one third of the patients found to be wheelchair bound at the time of the study. Disease severity, also expressed as the mean SPRS score, was 18.5/52 points (data available for 48 cases), whereas the cross-sectional progression rate, obtained by dividing the SPRS score by the disease duration in each individual, was on average 0.92 SPRS points per year (data available for 42 patients). Segregation analyses in families documented intrafamilial variability in terms of age at disease onset, clinical features and disease progression, as already illustrated by us and others (15, 16, 17).

### Molecular Findings

In this study, the known HSP-related *SPG* genes were analyzed, using a validated NGS TRP strategy, in a large cohort of patients. Over 95% of the bases in targeted regions showed an excellent quality value (QC >30) and >99% coverage of the targeted region with a read depth of at least 100X.

**Figure 2:**
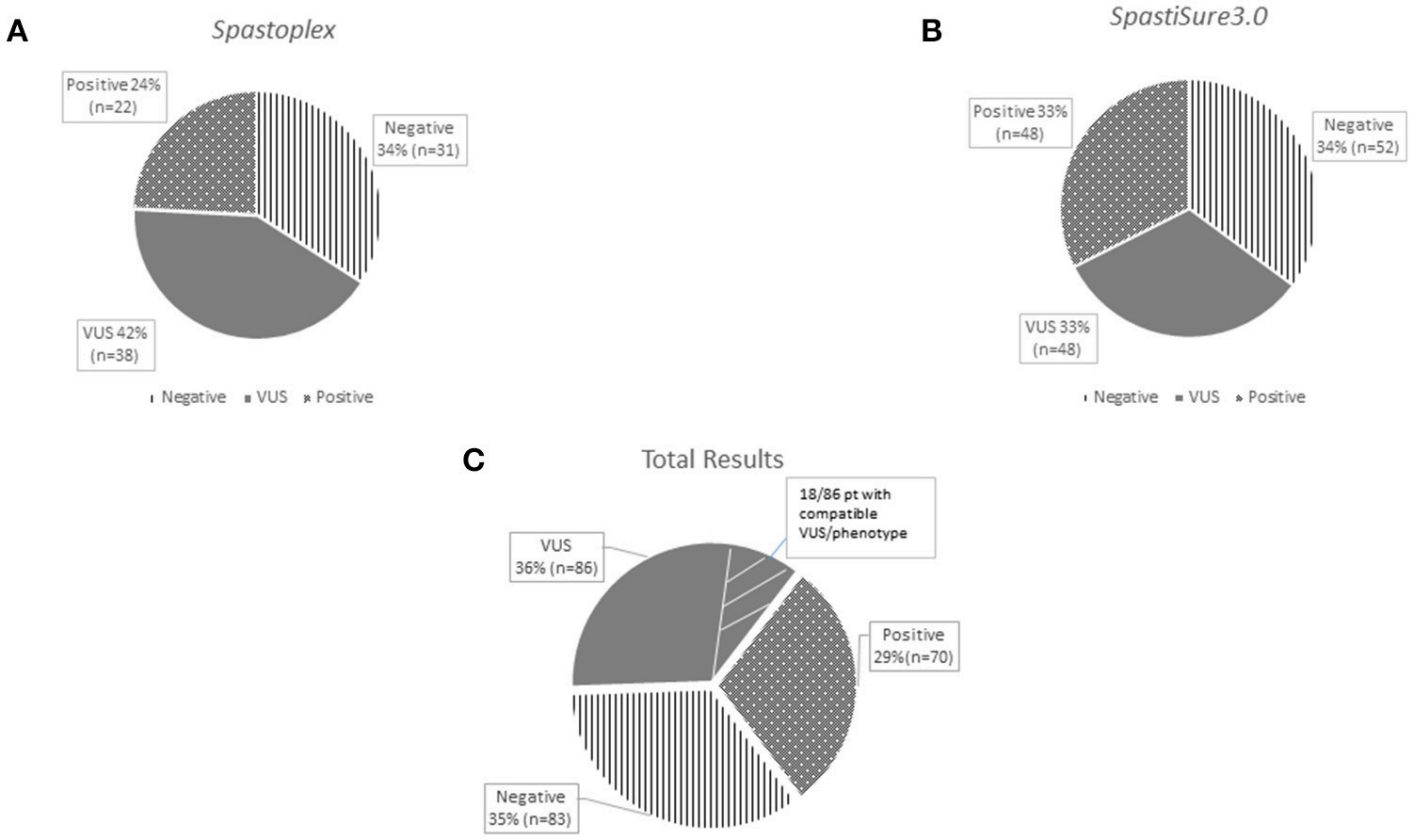
The graphs show the results obtained separately from the two panels used for the study: *Spastoplex* **(A)** and *Spastisure3.0* **(B)**. The dotted area indicates patients who received a genetic diagnosis; the gray area those found to harbor variants of unknown significance (VUS); and the striped area the cases that remained unsolved. The third graph **(C)** displays the final result of the cross-sectional study, obtained by averaging the data from the various panels. The VUS section contains a small striped segment, representing the patients (21%) found to carry a single variant in genes that, if mutated, can give rise to their phenotype.

*Spastoplex* was applied as a second- or third-line investigation in 91 index patients for molecular confirmation of HSP, whereas 148 patients underwent TRP analysis, with *SpastiSure3.0*, immediately, as a first-tier approach. Bioinformatic filtering and allele frequencies in public and internal databases were used to prioritize variant types. It was felt that critical, multicenter re-evaluation of clinical presentation, age at disease onset, and segregation in the families might help to establish whether variants could be annotated as disease associated. On the basis of such re-evaluation, and annotation of variants and their confirmation by Sanger sequencing, 85 known or probable pathogenic variants (12 homozygous and 73 heterozygous) were identified in a total of 70 cases, corresponding to a global positive diagnostic yield of 29% (Table 2). VUS were found in 86/239 patients (36% of the full cohort) and the majority of these were detected with the first TRP approach. Once we had adopted a more stringent bioinformatic filters than those described before by us and others (6), 83 patients (35% of the full cohort) were still without a molecular diagnosis (Figure 2) at the end of our study.

**Table 2:** List of mutations in 70 patients with confirmed diagnosis.

		**Coding**		**Protein**			
**Index case**	**Gene**	**NM_RefSeq**	**cDNA**	**NP_Ref**	**Protein**	**Allelic frequency**	**References**
Pt1	*CYP2U1*	NM_183075	c.1168C>T (hom)	NP_898898	p. Arg390Ter	1.219e-5	(18)
Pt2	*CPT1C*	NM_001199752	c.2133+1G>A (het)	NP_001186681	/	9.018e-6	This work
Pt3	*CYP7B1*	NM_004820	c.338insT (hom)	NP_004811	p.Phe114fsTer3	/	This work
Pt4	*DDHD1*	NM_001160148	c.1429C>T (hom)	NP_001153620	p.Arg477Ter	/	(19)
Pt5	*OPA1*	NM_130836	c.1180G>A (hom)	NP_570849	p.Ala394Thr	3.253e-5	(20)
Pt6	*FA2H*	NM_024306	c.1051A>G (het)	NP_077282	p.Ser351Gly	/	(21)
Pt6	*FA2H*	NM_024306	c.805C>T (het)	NP_077282	p.Arg269Cys	/	(21)
Pt7	*CYP7B1*	NM_004820	c.440_443delGCAAinsC (hom)	NP_004811	p.Gly147Aladel148Lys	/	This work
Pt8	*KIF1A*	NM_001244008	c.167A>G (het)	NP_001230937	p.Tyr56Ser	/	This work
Pt9	*KIF1C*	NM_006612	c.1046G>A (het)	NP_006603	p.Arg349His	1.082e-5	This work
Pt10	*PLP1*	NM_001128834	c.210T>G (het)	NP_001122306	p.Tyr70Ter	/	(22)
Pt11	*FA2H*	NM_024306	c.103G>T (het)	NP_077282	p.Asp35Tyr	1,22e-8	(23)
Pt11	*FA2H*	NM_024306	c.193C>T (het)	NP_077282	p.Pro65Ser	/	(21)
Pt12	*SPG11*	NM_025137	c.2833A>G (het)	NP_079413	p.Arg945Gly	3,12e-8	(24)
Pt12	*SPG11*	NM_025137	c.128delC (het)	NP_079413	p.Ser43fsTer15	/	This work
Pt13	*CYP7B1*	NM_004820	c.1108C>G (het)	NP_004811	p.Arg370Gly	/	This work
Pt13	*CYP7B1*	NM_004820	c.887A>G (het)	NP_004811	p.Asn296Thr	/	This work
Pt14	*DDHD2*	NM_015214	c.1978G>C (hom)	NP_056029	p.Asp660His	6.493e-5	(25)
Pt15	*CYP2U1*	NM_183075	c.343G>A (het)	NP_898898	p.Gly115Ser	6.625e-6	This work
Pt15	*CYP2U1*	NM_183075	c.1151G>T (het)	NP_898898	p.Arg384Ile	0.002300	This work
Pt16	*DDHD2*	NM_015214	c.759delT (hom)	NP_056029	p. Phe253fsTer13	/	This work
Pt17	*SPG7*	NM_003119	c.1A>T (het)	NP_003110	p.Metarg391Leu	1.146e-5	(26)
Pt18	*FA2H*	NM_024306	c.340_363del (het)	NP_077282	–	/	(21)
Pt18	*FA2H*	NM_024306	c.1055C>T (het)	NP_077282	p.Thr352Ile	/	(21)
Pt19	*KIF1A*	NM_001244008	c.760C>T (het)	NP_001230937	p.Arg254Trp	/	(27)
Pt20	*KIF1A*	NM_001244008	c.1048C>T (het)	NP_001230937	p.Arg350Trp	/	This work
Pt21	*WASHC5*	NM_014846	c.2504+1G>A (het)	NP_055661	/	/	This work
Pt22	*SPAST*	NM_014946	c.1625A>G (het)	NP_055761	p.Asp542Gly	0.0004141	(28)
Pt23	*CPT1C*	NM_001199752	c.1802C>T (het)	NP_001186681	p.Thr601Met	1.219e-5	This work
Pt24	*TRPV4*	NM_021625	c.1981C>T (het)	NP_067638	p.Arg661Cys	2.031e-5	This work
Pt25	*ERLIN2*	NM_007175	c.187C>A, (het)	NP_009106	p.Q63K	/	This work
Pt26	*WASHC5*	NM_014846	c.1924A>G (het)	NP_055661	p.Ile642Val	/	This work
Pt27	*KIF1A*	NM_001244008	c. 4927G>A (het)	NP_001230937	p.Asp1643Asn	0.0003241	This work
Pt27	*KIF1A*	NM_001244008	c.155T>C (het)	NP_001230937	p.Phe52Ser	/	This work
Pt28	*SPAST*	NM_014946	c.1245+4_1245+12delAGTGCTCTG (het)	NP_055761	–	/	This work
Pt29	*SPG7*	NM_003119	c.850_851delTTinsC (hom)	NP_003110	p.Phe284ProfsTer46	/	(26)
Pt30	*CAPN1*	NM_001198868	c.618_619delAG (hom)	NP_001185797	p.G208fsTer7	0,000005	This work
Pt31	*IFIH1*	NM_022168	c.1524+1G>T (het)	NP_071451	–	/	This work
Pt32	*SPG7*	NM_003119	c.1013G>T (het)	NP_003110	p.Gly338Val	/	This work
Pt33	*SETX*	NM_001351527	c.6122T>C (het)	NP_001338456	p.Ile2041Thr	0.0001383	This work
Pt34	*SPG11*	NM_025137	c.1203delA (het)	NP_079413	p.Lys401fsTer15	/	This work
Pt34	*SPG11*	NM_025137	c.6754+5G>A (het)	NP_079413	–	/	This work
Pt35	*L1CAM*	NM_000425	c.3628G>C (hom)	NP_000416	p.Asp1210His	5.653e-6	This work
Pt36	*SPAST*	NM_014946	c.323_328delTGCCGG (het)	NP_055761	p.V108_109del	/	This work
Pt37	*SPAST*	NM_014946	c.1496G>A (het)	NP_055761	p.Arg499His	/	(29)
Pt38	*SPAST*	NM_014946	c.1130G>A (het)	NP_055761	p. Gly377Glu	/	(30)
Pt39	*HSPD1*	NM_002156	c.188T>C (het)	NP_002147	p.Ile63Thr	/	This work
Pt40	*ERLIN2*	NM_007175	c.860_873dupAGGCCATTGCTTCC(hom)	NP_009106	–	/	This work
Pt41	*IFIH1*	NM_022168	c.1583T>G (het)	NP_071451	p.Leu528Arg	0.0004600	This work
Pt42	*SPAST*	NM_014946	c.164delA (het)	NP_055761	p.Tyr55fsTer5	/	This work
Pt43	*PNPLA6*	NM_001166111	c.3585C>G (het)	NP_001159583	p.Asp1195Glu	/	This work
Pt43	*PNPLA6*	NM_001166111	c.2389G>A (het)	NP_001159583	p.Val797Met	0.001776	This work
Pt44	*ADAR*	NM_001111	c.164C>T (het)	NP_001102	p.Pro55Leu	0.0001119	This work
Pt45	*DDHD1*	NM_001160148	c.2189dupT (het)	NP_001153620	p.Leu730fsTer23	/	This work
Pt45	*DDHD1*	NM_001160148	c.1503+6T>A (het)	NP_001153620	–	/	This work
Pt46	*GCH1*	NM_000161	c.510-1G>C (het)	NP_000152	–	/	This work
Pt47	*AP5Z1*	NM_014855	c.1302-1 G>T (het)	NP_055670	–	/	This work
Pt47	*AP5Z1*	NM_014855	c.2287G>A (het)	NP_055670	p.Val763Met	0.0001060	This work
Pt48	*ADAR*	NM_001111	c.2159T>C (het)	NP_001102	p.Val720Ala	2.165e-5	This work
Pt49	*ERLIN2*	NM_007175	c.502G>A (het)	NP_009106	p.Val168Met	/	This work
Pt50	*BICD2*	NM_001003800	c.793A>G (het)	NP_001003800	p.Met265Val	2.525e-5	This work
Pt51	*ABCD1*	NM_000033	c.836T>C (het)	NP_000024	p.Leu279Pro	/	(31)
Pt52	*DDHD2*	NM_015214	c.38delA (het)	NP_056029	p.Gln13fsTer16	/	This work
Pt52	*DDHD2*	NM_015214	c.864A>C (het)	NP_056029	p.Ile288Ile	0.0003393	This work
Pt53	*GCH1*	NM_000161	c.454-2A>T (het)	NP_000152	–	/	This work
Pt54	*ZFYVE27*	NM_001002261	c.149A>G (het)	NP_001002261	p.Tyr50Cys	3.968e-5	This work
Pt55	*SPAST*	NM_014946	c.1728+1G>A (het)	NP_055761	–	/	(32)
Pt56	*SPG11*	NM_025137	c.6754+5G>A (hom)	NP_079413	–	/	This work
Pt57	*ABCD1*	NM_000033	c.1165C>T (emi)	NP_000024	p.Arg389Cys	4.634e-5	(33)
Pt58	*SETX*	NM_001351527	c.3992C>T (het)	NP_001338456	p.Pro1331Leu	0.0004152	This work
Pt59	*REEP1*	NM_001164730	c.54-2A>G (het)	NP_001158202	–	/	This work
Pt60	*TUBB4A*	NM_001289129	c.1072C>T (het)	NP_001276058	p.Pro358Ser	/	This work
Pt61	*REEP1*	NM_001164730	c.324 + 1G>A (het)	NP_001158202	–	/	This work
Pt62	*WASHC5*	NM_014846	c.1550A>G (het)	NP_055661	p.Asn517Ser	2.922e-5	This work
Pt63	*POLR3A*	NM_007055	c.1031G>T (het)	NP_008986	p.Arg344Leu	/	This work
Pt63	*POLR3A*	NM_007055	c.1909+22G>A (het)	NP_008986	–	0,00001	(34)
Pt64	*SPAST*	NM_014946	c.1456A>G (het)	NP_055761	p.Thr486Ala	/	This work
Pt65	*ATL3*	NM_015459	c.758T>C (het)	NP_056274	p.Ile253Thr	/	This work
Pt66	*POLR3A*	NM_007055	c.3201_3202delGC (het)	NP_008986	p.Arg1069fsTer2	/	This work
Pt66	*POLR3A*	NM_007055	c.1909+22 G>A (het)	NP_008986	–	0,00001	(34)
Pt67	*KIF1A*	NM_001244008	c.460G>T (het)	NP_001230937	p.Val154Phe	/	This work
Pt68	*MFN2*	NM_014874	c.2183A>G (het)	NP_055689	p.Gln728Arg	/	This work
Pt69	*CYP7B1*	NM_004820	c.1362dupT (het)	NP_004811	p.Ala455CysfsTer17	4.063e-6	This work
Pt69	*CYP7B1*	NM_004820	c.344 C>T (het)	NP_004811	p.Ser115Phe	4.106e-6	This work
Pt70	*SPG11*	NM_025137	c.5014G>T (het)	NP_079413	p.Glu1672Ter	/	(35)
Pt70	*SPG11*	NM_025137	c.3122_3124delGAC (het)	NP_079413	p.Arg1041del	/	This work

**Figure 3:**
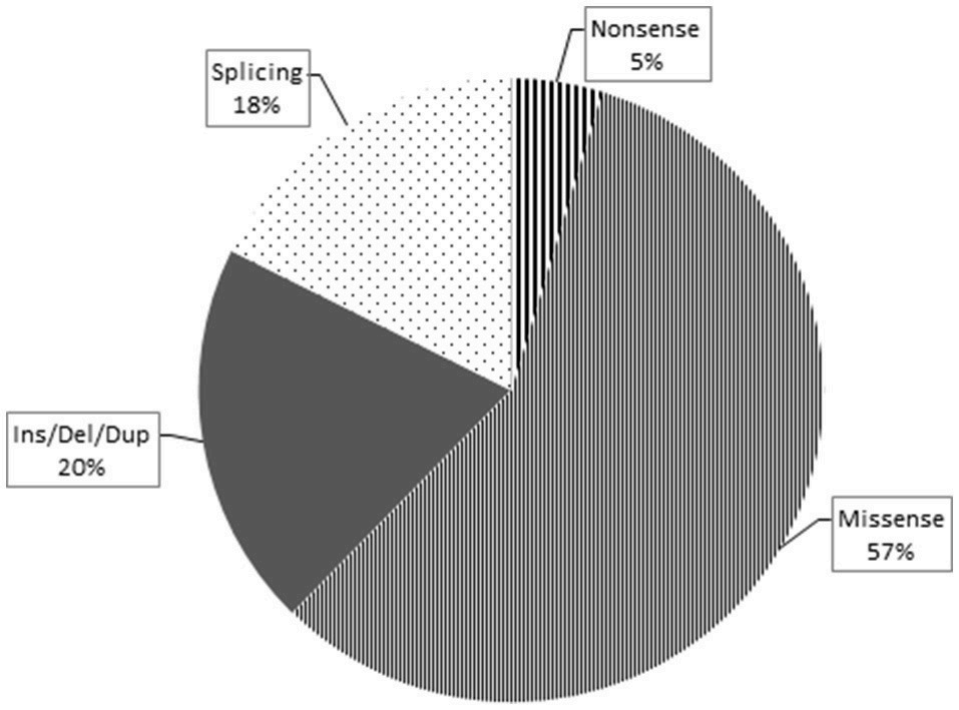
This pie chart shows the different mutation types and their relative frequency among the pathogenic ones identified in this study. As expected, the missense type (thin stripes) is the most frequent, followed by the Ins/Del/Dup (dark gray), splicing (dots), and finally nonsense (thick stripes) types.

**Figure 4:**
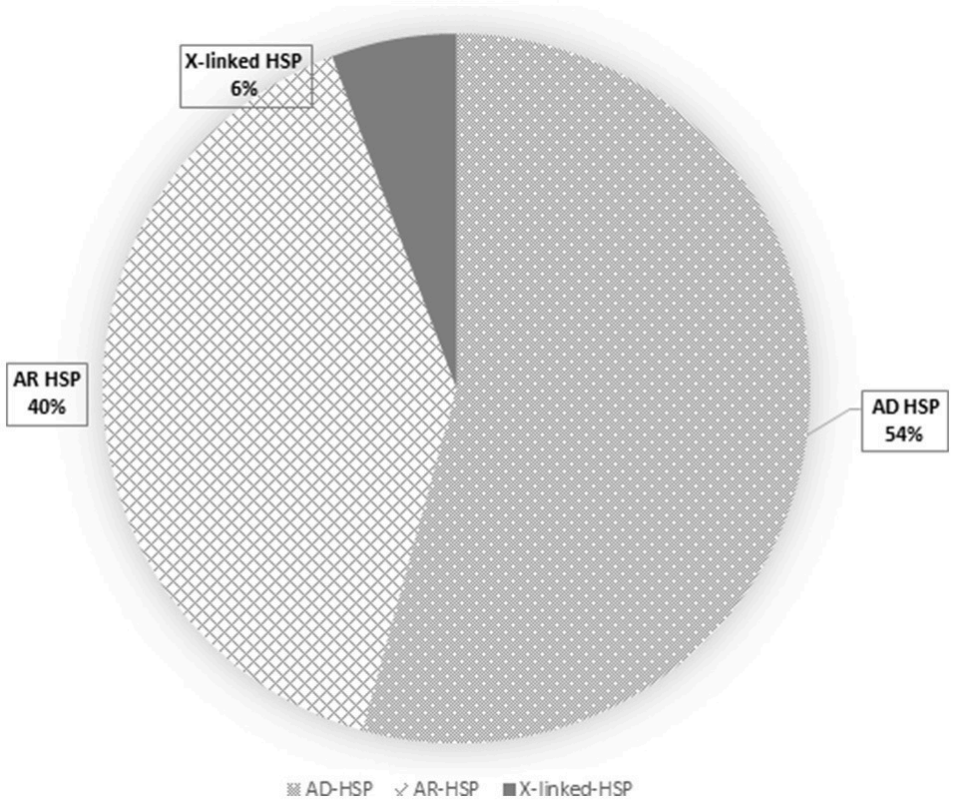
This pie chart shows the frequency of HSP inheritance patterns: autosomal dominant HSP is the most frequent (dots), followed by the autosomal recessive (squares) and the X-linked (dark gray) forms.

Overall, loss-of-function mutations accounted for 43% and missense mutations for 57% of the variants detected (Figure 3). Only four mutations causing a premature stop codon were identified, whereas no large deletions/duplications were detected. As regards the patterns of inheritance among the diagnosed patients, 54% (38/70) had a documented autosomal dominant (AD) pattern of transmission, while 40% (28/70) presented either AR HSP or were sporadic cases in which AR inheritance had not previously been demonstrated. The remaining four cases had X-linked HSP (Figure 4).

**Figure 5:**
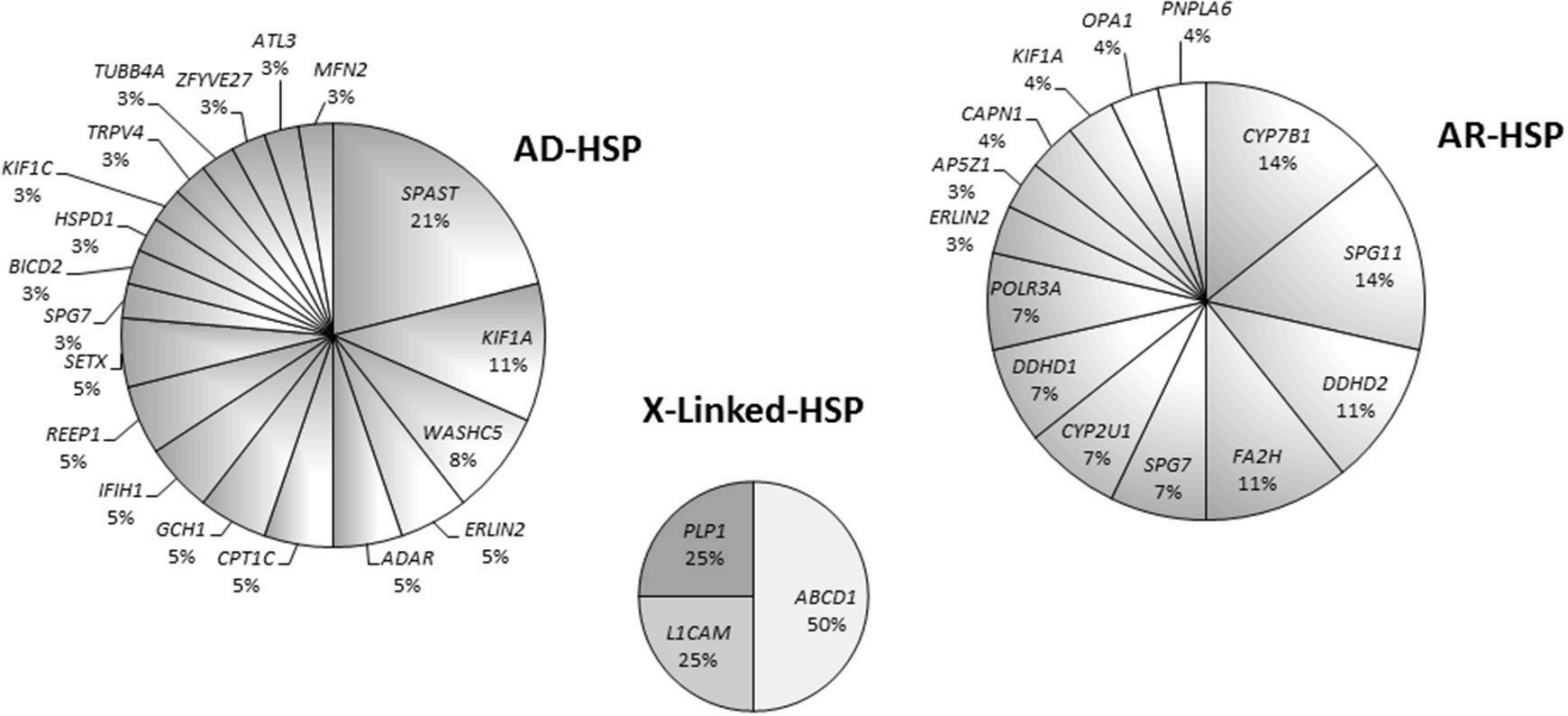
Pie charts showing gene mutation rates by pattern of inheritance in 70 patients.

Eight patients harbored mutations in *SPAST* (8/38), which appeared to be the most common mutated AD gene identified in our study. Other mutated AD genes were considerably rarer. In our cohort, about half of the mutations in AR HSP genes occurred in four genes, namely *KIAA1840/*SPG11, *FA2H/*SPG35, *CYP7B1/*SPG5, and *DDHD2/*SPG54 (Figure S1). In the X-linked forms, two patients carried different missense variants in *ABCD1*, one had a missense variant in *L1CAM* /SPG1, and a single case harbored a nonsense change in *PLP1/*SPG2 (Figure 5).

Interestingly 26/70 patients (36%) with a clear molecular diagnosis of HSP also showed VUS in other genes, especially *KIAA1840/*SPG11 and *SACS*. However, *STRING* analysis of these cases did not reveal obvious protein-to-protein interactions that might have partly explained a more complex genotype (data not shown).

Overall, most VUS occurred in a set of four genes, namely *KIAA1840/*SPG11, *SACS, AP5Z1*/SPG48 and *LYST* (Figure S2). Among the 86 patients presenting VUS, we enlisted 18 (21%) who are still under investigation because they harbor a single variant in at least one gene that, if mutated, is known to give rise to the clinical conditions they display (Figure 2C). However, since we lack complete CRF data for these cases and/or segregation analysis could not be performed in them, patients were considered *bona fide* members of the VUS group in spite of lacking a complete molecular study. The other 68 HSP cases with VUS showed no molecular findings correlating directly with their clinical features; however, it cannot be excluded that some of the genetic changes they show, as well as new (as yet unknown) genetic changes, play a role in causing the phenotype. Eighty-three index cases resulted molecularly undefined in our NGS study (35% negative yield). Full dataset of gene variants generated for this study can be found in the ClinVar genetic repository (https://www.ncbi.nlm.nih.gov/clinvar/) (submission code SUB4568040).

## Discussion

The wide clinical and genetic heterogeneity of HSP and the increasingly thin line between the various ataxic-spastic and spastic-dystonic forms mean that it is still difficult to arrive at a rapid and precise diagnosis of this condition. NGS approaches are increasingly being used for genetic diagnostics in routine clinical settings, and different published papers report successful use of this technology in HSP (36–39), with positive yields ranging from 20% in adult cases to 52.5% in child cohorts (3, 39–43). To our knowledge, the present cross-sectional study of 239 HSP cases is among the largest in Europe and Italy to have used TRP analysis. The present study, which addressed “many genes in many patients” and investigated practically all the known genes related to HSP and similar motor neuron disorders, was expected to give a higher positive diagnostic yield than it actually did; furthermore, at least 60% of the patients were investigated underwent NGS testing as a first-tier approach, in order to reflect modern procedures in diagnostic laboratories. However, initial expectations notwithstanding, the overall findings showed a 29% diagnostic yield, a rate similar to those reported in other studies, including more recent ones that present population-specific data (44, 45). There are two possible reasons for this unexpected result. First, the molecular criteria and filtering options used in the present study to define positive cases were perhaps too stringent. Had we used looser criteria and disregarded absence of clinical and segregation data, we would have identified 18 additional cases. Second, about 20 further patients who harbored single potential pathogenic mutations were classified as VUS, since we did not specifically look for second mutations. For example, in two cases we identified, respectively, the p.Ala510Val mutation in *SPG7*, which is the most common alteration of the paraplegin gene (46), and a nonsense variant in *FA2H*/SPG35, but did not look for deep intronic changes or gross genomic rearrangements by additional methodologies such as customized array-CGH. Moreover, we decided to exclude variants with >1 homozygous count in gnomAD even those predicted to be deleterious and reported in the Human Gene Mutation Database (HGMD, www.hgmd.cf.ac.uk/). Thus, we can reasonably assume that our diagnostic yield is at least 10–15% lower than the true rate, and therefore can hardly be considered a complete molecular picture of the cohort. The higher yield we obtained when using TRP for first-tier analysis could be attributed to the fact that our collaborators' clinical data collection became more precise and complete once we had adopted common criteria for inclusion in the study (a common CRF), or it may indicate that new TRP designs offer improved “genotypability,” or even both. Our study is offering results in line with current literature of diagnostic power in HSP (see Table 3). Previous studies have adopted TRP methods (either alone or followed by WES analyses) to corroborate a clinical diagnosis of HSP and the range of diagnostic yield varied from 19 to 62% (36, 39, 41–44, 47–52).

**Table 3:** Relative frequency of diagnostic yield in NGS analyses of patients with hereditary spastic paraplegia.

**NGS method**	**Genes analyzed**	**Number of patients**	**Diagnostic rate (%)**	**References**
TRP	16	31	15/31 (48%)	(39)
TRP	60	42	13/51 (25%)^a^	(36)
TRP	12	29	14/29 (48%)	(42)
TRP (I)	34	25	8/25 (32%)	(44) (total 20%)
TRP (II)	70	73	12/73 (16%)
TRP	159	105	20/105 (19%)^b^	(43)
TRP	113	47	29/47 (62%)	(47)
TRP	62	55	34/55 (62%)	(48)
TRP	149	99	47/99 (47%)	(49)
TRP + WES	58	97	25/97 (26%)	(41)
TRP+WES	51	37	150/526 (28.5%)	(50)
Clinical exome	2,731	9	6/9 (67%)	(39)
Clinical exome	4,813	66	18/66 (27%)	(40)
Clinical exome	unknown	48	8/48 (17%)^c^	(45)
WES	/	9	13/51 (25%)^a^	(36)
WES	/	12	6/12 (50%)	(51)
WES	/	21	13/21 (62%)	(52)

*TRP, targeted resequencing panel; WES, whole exome sequencing*.

a considering the whole cohort of patients;

b 29%, if also variants of unknown significance were considered;

c *33%, considering also probably causative variants*.

The study we presented also identified a large set of novel variants (see Table 2): indeed, < 40% of the 85 pathogenic mutations detected in this research had previously been reported in the literature, or are listed in HGMD. These findings therefore further corroborate the larger-than-expected allelic heterogeneity of the Italian HSP population previously reported in the literature. We confirmed that mutations in *SPAST*/SPG4 account for more than 20% of solved cases, followed by other relatively less common AD *SPG* genes (namely, *KIF1A* and *WASHC5*). It is of note that we found few variants in *REEP1*/SPG31 but did not detect mutations in *ATL1*/SPG3A, which has previously been reported to be the second most common genes responsible for AD HSP, and among those frequently involved in early-onset pure forms (53). However, in our cohort only 16 patients developed pure HSP before the age of 10 years and they were all studied using *Spastoplex* where involvement of *ATL1/*SPG3 had been ruled out by others before their inclusion in the present study. With regard to the recessive forms, our study confirmed that *SPG11* is one of the most common AR HSP genes: indeed, together with those in *CYP7B1*/SPG5, *DDHD2*/SPG54, and *FA2H*/SPG35, mutations in *SPG11* account for half of all AR cases. Finally, the relatively low frequency of mutations in *SPG7* might be related to their study in a concurrent “ataxia-associated” NGS panel (54).

It is worth noting that our data lend support to the notion that zygosity is no longer a barrier to defining a molecular diagnosis in HSP. We detected at least three genes (*ERLIN2, KIF1A* and *SPG7*) in which mutations could be inherited in an either dominant or recessive fashion, suggesting that an even more HSP genes might actually have different modes of inheritance. Were this found to be the case, it would have implications for the estimated diagnostic yield in real-life molecular diagnostic settings and could point to differences in disease-associated phenotypes. Importantly, we found unusual clinical manifestations in some cases. We detected two families with the same mutation in *POLR3A*; in one patient this was found to be associated with pure HSP, and in another with HSP plus optic atrophy and sensory ataxia. In another family, we found slight motor involvement, cataract, cluster headache and lower limb motor impairment in a mother, and lower motor neuron disease in a grandmother, as well as stiff legs, urinary urgency, and slight cerebellar involvement with bipolar disorder in a daughter harboring the same mutation in *KIF1A*. Given that we are expanding the ways in which we genotype HSP patients, we should no longer be surprised to encounter an increasing variety of features associated with the condition.

Finally, it is worth dwelling on one particular result emerging from this study. Around 36% of the patients studied presented VUS, and were therefore left with molecularly undefined or uncertain diagnoses. Clinical manifestations in this group (see Table S2). we're not significantly different from the index cases with a molecular definition. Various technical limitations of the present study, the failure to investigate large gene rearrangements or regulatory intron regions, and even the rare presence of mosaicisms are all factors that might account for these incomplete diagnoses. Alternative explanations, such as very rarely, different zygosity for a known gene, the presence of clinical phenocopies, or the involvement of new HSP genes yet to be discovered, can also apply to this group of patients, as well as to the 83 index cases who were undiagnosed. Nonetheless, we feel that this apparent “missing heritability” in HSP is unlikely to be attributable solely to mutations in as yet unidentified genes. New genes emerged in the past 4 years account for less of 1% of the unsolved cases. It is far more likely that mutations not amenable to current standard approaches including structural variants and variants outside of annotated coding exons account for a substantial share of unexplained cases. Although full use of exome sequencing in clinical diagnosis is an option (44), we therefore propose that the next TRP to be designed should consider complete gene regions and use better bioinformatics tools in order to detect structural variants and uncover the significance of changes in regulatory regions.

Considering the limitations of our study, the increasing clinical overlap between the neurogenetic conditions in different motor neuron diseases (4), and the still large number of HSP patients who remain unsolved, it should be asked whether modern NGS approaches in clinical diagnostic laboratories should switch to more comprehensive technologies. Whilst genome sequencing is increasingly “knocking at the door” of routine clinical diagnostic practice, most national health systems cannot afford to implement this technique fully. In this setting, exome analyses seem to be a practical option. Use of clinical exome sequencing has shown its value in guiding practical patient management, and its diagnostic yield, ranging from 16 to 40% (5, 36), is similar to that of TRP, though reimbursements of costs is not similarly obvious in most health services. Furthermore, coding exon sequencing appears to be a feasible as first-tier diagnostic approach in naive HSP patients. It also seems reasonable to speculate that only full genome studies, or a combination of whole-exome sequencing with RNA studies, are likely to increase diagnostic yield beyond the present rates, once their costs are more affordable on a large scale.

## Conclusion

The advent of the NGS technique in the last decade has revolutionized the way we approach neurogenetic diagnoses in general, and the diagnosis of HSP in particular. The results of the present study show that the TRP method, used as the first step in clinical diagnostic laboratories, was able to provide information of real clinical significance in about 30–40% patients tested, and it emerged as a relatively inexpensive option (< 200 euros per sample). Deeper phenotyping of patients in the clinic, integrated with more rapid use of functional validation (*in vitro* or *in silico*), should be mandatory, whether one prefers to use exome sequencing or, instead, larger TRPs, investigating beyond the coding exons. The development of new and more precise sequencing tools combined with universal data sharing, such as multinational initiatives as in GENESIS2.0 (55), and stringent bioinformatics criteria, could also be helpful for annotating variants, especially those classified as VUS. In the era of medical genomics and precision medicine, which has brought the first randomized clinical trials in HSP (56) and a deeper approach in modern neurorehabilitation (57), high levels of genetic heterogeneity should no longer prolong the time to diagnosis and preclude access to new treatment and care opportunities.

## Ethics Statement

This study was carried out in accordance with the recommendations of Regione Toscana Ethic Committee with written informed consent from all subjects. All subjects gave written informed consent in accordance with the Declaration of Helsinki. The protocol was approved by the Regione Toscana Ethic Committee.

## Author Contributions

AD drafted the manuscript and critically revised molecular results. AD, AT, and MB performed molecular studies and bioinformatics analyses. CaC, MD, AR, GS, RB, CB, IB, CrC, CD, GD, VD, MF, NF, CF, SG, GG, CG, RG, FiG, AK, ML, ChaM, AlM, PM, FM, LM, MM, RM, OM, EP, APe, APi, FP, MP, MS, FrS, FT, AT, MV, AA, GA, ChrM, AM, AlP, IR, and SR Contributed clinical information, assessed severity score, and contributed significantly to genotype/phenotype correlations. AlF and AnF revised the clinical strategy and edited the manuscript. FeG and FiS conceived the study, oversaw data acquisition, directed genotype/phenotype correlations, supervised the initial draft and critically revised the manuscript.

### Conflict of Interest Statement

The authors declare that the research was conducted in the absence of any commercial or financial relationships that could be construed as a potential conflict of interest.

## Supplementary Material

Figure S1 Histogram listing mutated genes and the numbers of cases harboring each mutation, starting from the least frequently mutated genes (*n* = 1) to the most commonly mutated one, *SPAST* (*n* = 8).

Figure S2 Histogram listing genes with variants of unknown significance and the numbers of cases harboring each one, starting from the least frequently mutated (*n* = 1) to the most commonly mutated ones (*SPG11,n* = 11*; SACS* and *AP5Z1*, each, *n* = 9*; LYST, n* = 8).

Table S1 List of genes associated with hereditary spastic paraplegia and included in the targeted resequencing gene panel.

Table S2 Main clinical features in patients molecularly undefined.
