# Supplementary figures and images for: Next Generation Molecular Diagnosis of Hereditary Spastic Paraplegias: An Italian Cross-Sectional Study
Source: Front Neurol. 2018 Dec 4;9:981. doi: 10.3389/fneur.2018.00981 (PMC6289125; doi:10.3389/fneur.2018.00981)

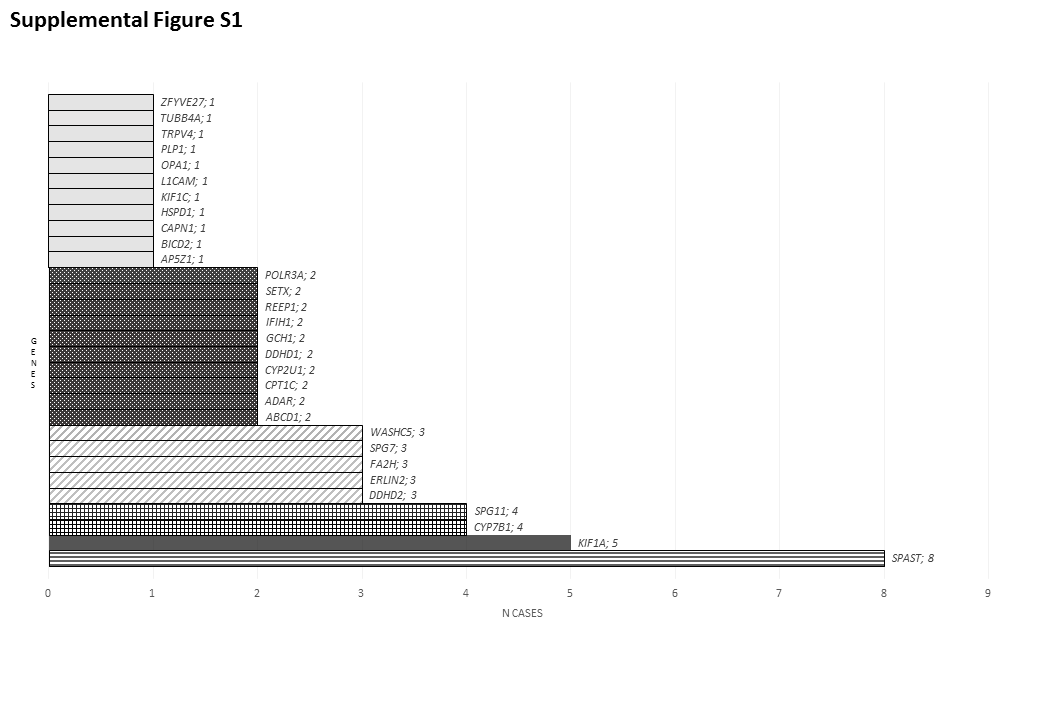

Supplement: Figure S1 — Histogram listing mutated genes and the numbers of cases harboring each mutation, starting from the least frequently mutated genes (n = 1) to the most commonly mutated one, SPAST (n = 8). [file Image_1.TIF]

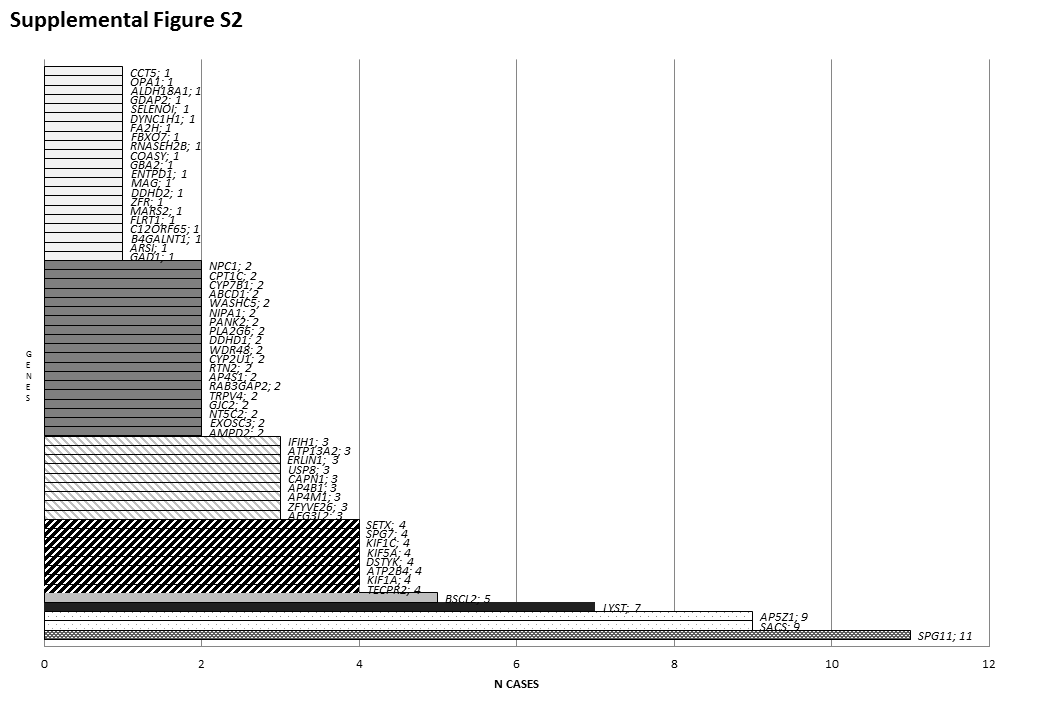

Supplement: Figure S2 — Histogram listing genes with variants of unknown significance and the numbers of cases harboring each one, starting from the least frequently mutated (n = 1) to the most commonly mutated ones (SPG11,n = 11; SACS and AP5Z1, each, n = 9; LYST, n = 8). [file Image_2.TIF]
