# Supplementary material for: Next Generation Molecular Diagnosis of Hereditary Spastic Paraplegias: An Italian Cross-Sectional Study
Source: Front Neurol. 2018 Dec 4;9:981. doi: 10.3389/fneur.2018.00981 (PMC6289125; doi:10.3389/fneur.2018.00981)
Supplement: Table S1 — List of genes associated with hereditary spastic paraplegia and included in the targeted resequencing gene panel. [file Table_1.DOCX]

**Supplemental Table S1.** List of genes associated with hereditary spastic paraplegia and included in the targeted resequencing gene panel

| **Gene** | **RefSeq** |
| --- | --- |
| *ABCD1* | NM_000033 |
| *ACBD5* | NM_001352572 |
| *ADAR* | NM_001111 |
| *AFG3L2* | NM_006796 |
| *ALDH18A1* | NM_001323412 |
| *ALS2* | NM_020919 |
| *AMPD2* | NM_004037 |
| *AP4B1* | NM_004037 |
| *AP4E1* | NM_007347 |
| *AP4M1* | NM_004722 |
| *AP4S1* | NM_007077 |
| *AP5Z1* | NM_014855 |
| *ARL6IP1* | NM_015161 |
| *ARSI* | NM_001012301 |
| *ATAD3A* | NM_018188 |
| *ATL1* | NM_015915 |
| *ATL3* | NM_015459 |
| *ATP2B4* | NM_001684 |
| *ATP13A2* | NM_022089 |
| *B4GALNT1* | NM_001478 |
| *BICD2* | NM_001003800 |
| *BSCL2* | NM_001122955 |
| *C12ORF65* | NM_152269 |
| *C19ORF12* | NM_001031726 |
| *C9ORF72* | NM_001256054 |
| *CAPN1* | NM_001198868 |
| *CCT5* | NM_012073 |
| *COASY* | NM_001042532 |
| *CPT1C* | NM_001199752 |
| *CYP27A1* | NM_000784 |
| *CYP2U1* | NM_183075 |
| *CYP7B1* | NM_004820 |
| *DARS2* | NM_018122 |
| *DDHD1* | NM_001160148 |
| *DDHD2* | NM_015214 |
| *DSTYK* | NM_015375 |
| *DYNC1H1* | NM_001376 |
| *ENTPD1* | NM_001164178 |
| *EPT1* | NM_033505 |
| *ERLIN1* | NM_001100626 |
| *ERLIN2* | NM_007175 |
| *EXOSC3* | NM_016042 |
| *EXOSC8* | NM_181503 |
| *FA2H* | NM_024306 |
| *FARS2* | NM_001318872 |
| *FBXO7* | NM_012179 |
| *FLRT1* | NM_013280 |
| *GAD1* | NM_000817 |
| *GBA2* | NM_020944 |
| *GCH1* | NM_000161 |
| *GDAP1* | NM_018972 |
| *GDAP2* | NM_017686 |
| *GJA1* | NM_000165 |
| *GJC2* | NM_020435 |
| *GLB1* | NM_000404 |
| *HSPD1* | NM_002156 |
| *IBA57* | NM_001010867 |
| *IFIH1* | NM_022168 |
| *KCNA2* | NM_004974 |
| *KIAA0196* | NM_014846 |
| *KIF1A* | NM_001244008 |
| *KIF1C* | NM_006612 |
| *KIF5A* | NM_004984 |
| *KY* | NM_178554 |
| *L1CAM* | NM_000425 |
| *LYST* | NM_000081 |
| *MAG* | NM_002361 |
| *MARS* | NM_004990 |
| *MARS2* | NM_138395 |
| *MFN2* | NM_014874 |
| *MTHFR* | NM_005957 |
| *NIPA1* | NM_144599 |
| *NPC1* | NM_000271 |
| *NT5C2* | NM_001134373 |
| *OPA1* | NM_130836 |
| *OPA3* | NM_001017989 |
| *PANK2* | NM_153638 |
| *PEX16* | NM_057174 |
| *PGAP1* | NM_001321099 |
| *PLA2G6* | NM_001349864 |
| *PLP1* | NM_001128834 |
| *PNPLA6* | NM_001166111 |
| *POLR3A* | NM_007055 |
| *RAB3GAP2* | NM_012414 |
| *REEP1* | NM_001164730 |
| *REEP2* | NM_001271803 |
| *RNASEH2B* | NM_024570 |
| *RTN2* | NM_005619 |
| *SACS* | NM_001278055 |
| *SAMHD1* | NM_015474 |
| *SERAC1* | NM_032861 |
| *SETX* | NM_001351527 |
| *SLC16A2* | NM_006517 |
| *SLC33A1* | NM_001190992 |
| *SOX10* | NM_006941 |
| *SPAST* | NM_014946 |
| *SPG11* | NM_025137 |
| *SPG20* | NM_001142295 |
| *SPG21* | NM_016630 |
| *SPG7* | NM_003119 |
| *TECPR2* | NM_014844 |
| *TFG* | NM_001195478 |
| *TRMT5* | NM_001350254 |
| *TRPV4* | NM_021625 |
| *TTC19* | NM_001271420 |
| *TUBB4A* | NM_001289129 |
| *UBQLN2* | NM_013444 |
| *UCHL1* | NM_004181 |
| *USP8* | NM_001128610 |
| *VAMP1* | NM_014231 |
| *VCP* | NM_007126 |
| *VPS37A* | NM_152415 |
| *VRK1* | NM_003384 |
| *WDR48* | NM_001303402 |
| *ZFR* | NM_016107 |
| *ZFYVE26* | NM_015346 |
| *ZFYVE27* | NM_001002261 |
