# Supplementary material for: Next Generation Molecular Diagnosis of Hereditary Spastic Paraplegias: An Italian Cross-Sectional Study
Source: Front Neurol. 2018 Dec 4;9:981. doi: 10.3389/fneur.2018.00981 (PMC6289125; doi:10.3389/fneur.2018.00981)
Supplement: Table S2 — Main clinical features in patients molecularly undefined. [file Table_2.DOCX]

**Supplemental Table S2.** Main clinical features in patients molecularly undefined

__________________________________________________________________

M/F 41/42

Familial/Sporadic 23/83

Dominant/Recessive/X-linked 6/14/3

Age at onset, y, mean ± SD (n) 19.5±15.4 (54)

Duration, mean ± SD (n) 11.3±2.2 (39)

Disability, mean ± SD (n) 2.1±1.3 (33)

SPRS, average score (n) 16.0 (33)

Disability

Stick use 59.2 %

Wheelchair use 24.7 %

Upper limb

Hypertonia 16.5 %

Hyperreflexia 14.5 %

Lower limb

Abnormal vibration sense 74.3 %

Amyotrophy 7.5 %

*Pes cavus* 24.6 %

Bilateral clonus 71.4 %

Urinary dysfunction 57.5 %

Abnormal MEP

lower limbs 73.2 %

upper limbs 59.5 %

MRI abnormalities 64 %

Cerebellar atrophy 21 %

Hyperintense WM 14 %

Thin corpus callosum 11 %

_________________________________________________________

SPRS= Spastic Paraplegia Rating Scale; WM=white matter
